# MicroRNA-129-2-3p directly targets Wip1 to suppress the proliferation and invasion of intrahepatic cholangiocarcinoma

**DOI:** 10.7150/jca.41492

**Published:** 2020-03-05

**Authors:** Chen Chen, Jinqiong Jiang, Meng Fang, Lei Zhou, Yongzhi Chen, Jia Zhou, Yinghui Song, Gaoying Kong, Bao Zhang, Bo Jiang, Hao Li, Chuang Peng, Sulai Liu

**Affiliations:** 1 Department of Hepatobiliary Surgery, Hunan Provincial People's Hospital/The First Affiliated Hospital of Hunan Normal University, Changsha, Hunan Province, People's Republic of China.; 2 Department of Oncology, Hunan Provincial People's Hospital/The First Affiliated Hospital of Hunan Normal University, Changsha, Hunan Province, People's Republic of China.; 3 Department of Anesthesiology, Hunan Provincial People's Hospital/The First Affiliated Hospital of Hunan Normal University, Changsha, Hunan Province, People's Republic of China.; 4 Clinical Research Center for Anesthesiology of ERAS in Hunan Province, Changsha 410005, China.; 5 Department of Minimally Invasive Surgery, The Second People's Hospital of Hunan Province, Changsha 410017, China.

**Keywords:** miR-129-2-3p involved ICC via Wip1

## Abstract

Accumulated studies showed that numerous microRNAs (miRNAs) were aberrantly expressed in human intrahepatic cholangiocarcinoma (ICC) and contributed to the tumorigenic processes. However, whether miR-129-2-3p is implicated in the ICC initiation and progression is still limited. Here, the results revealed that miR-129-2-3p expression was notably decreased in ICC tissues and cell lines, and that a low miR-129-2-3p expression was obviously associated with distant metastasis and clinical stage. Exogenous miR-129-2-3p expression evidently repressed the proliferative and invasive abilities of ICC cells. Mechanistic studies indicated that Wild-type p53-induced phosphatase 1 (Wip1) was a direct target gene for miR-129-2-3p in ICC cells. Furthermore, silencing Wip1 expression mimicked the suppressive effects of miR-129-2-3p upregulation on ICC cells. Interestingly, reintroduction of Wip1 expression partially abolished the miR-129-2-3p -reduced cell proliferation and invasion in ICC. Moreover, ectopic miR-129-2-3p expression hindered the ICC tumor growth *in vivo*. To the best of our knowledge, it is the first time to reveal that miR-129-2-3p plays a crucial role in tumor suppression in ICC pathogenesis through directly targeting Wip1. These results will aid in elucidating the roles of miR-129-2-3p in ICC, and suggest that this miRNA may provide a potential target for the treatment of ICC

## Introduction

Intrahepatic cholangiocarcinoma (ICC) is an aggressive and poorly understood biliary malignancy that is frequently diagnosed at advanced stages, which limits its treatment options 1. In recent years, the incidence and mortality of ICC has drastically increased with geographic variation 2,3. Furthermore, the long-term survival of patients with unresectable ICC is dismal, with less than 5% to 10% of patients surviving at 5 years after the diagnosis 2,4. Surgical resection remains the only approach with curative intent, achieving a 5-year survival rate of approximately 20% at early T1-T2 stages; Still worse, in sharp contrast to the use of standard therapies for patients with advanced lung/breast/colorectal cancers and hepatocellular carcinoma, systemic chemotherapy and molecular-targeted therapies have had limited success in treating ICC 5. Therefore, it is imperative to fully elucidate the mechanisms responsible for ICC carcinogenesis and progression, as they may provide novel and effective strategic approaches to improve the prognosis of ICC patients.

Recently, microRNAs was shown to implicated in controlling multiple steps of ICC onset and progression 6. Aberrant expression of the miR-129-2-3p has been detected in multiple types of carcinoma, yet its expression and potential biologic role in ICC has not been investigated 7-9.

Wip1 (also known as PPM1D) is a monomeric serine/threonine phosphatase of the PP2C family, and its expression is increased after DNA damage, which inactivates p53 and promotes termination of the DDR pathway 10,11. Wip1 dephosphorylates many proteins, including ataxia-telangiectasia mutated (ATM), Chk1, Chk2, p53, p38 and Mdm2 10. These proteins that belong to DNA damage response markers are often decreased in DNA damage response pathways, which contribute to tumor progression. Others and our previous studies showed that Wip1 were over expression in a wide range of tumor tissues including kidney 12, breast 13, lung 14, ovarian 15, neuroblastoma 16. Our previous research showed that Wip1 was highly expressed in ICC tissues and cell lines and may be a key regulator in the tumorigenicity and invasion of human ICC 17,18. However, the potential mechanism is unknown. In current study, we decided to investigate the effects of miR-129-2-3p in ICC and have demonstrated that miR-129-2-3p could inhibit cell invasion and migration via Wip1 in ICC.

## Materials and methods

### Clinical tissue specimens

In total, 90 patients with ICC who received surgical excision in Hunan Provincial People's Hospital/The First Affiliated Hospital of Hunan Normal University were recruited between Autumn 2016 and May 2017. None of the patients received any anticancer therapies before surgery, including chemotherapy, radiotherapy or immunotherapy. Following surgical resection, tumor specimens were quickly frozen in liquid nitrogen and then stored at -80 °C. Prior written informed consent was provided from all participants and this study was approved by the Ethics Committee of Hunan Provincial People's Hospital/The First Affiliated Hospital of Hunan Normal University.

### Cell culture and transient transfection

A panel of human ICC cell lines, including QBC-939, RBE, as well as a normal human biliary epithelial cell (BEC) were purchased from the Type Culture Collection of the Chinese Academy of Sciences (Shanghai, China). Cells were incubated under the conditions of 5% CO_2_ and 37°C in Dulbecco's modified Eagle's medium (DMEM) containing 10% fetal bovine serum (FBS; both from Gibco, Invitrogen, Carlsbad, CA, USA) and 1% penicillin/ streptomycin mixture (Sigma‐Aldrich, St. Louis, MO).

The synthetic miR-129-2-3p mimics and miRNA mimics negative control (miR-NC) were obtained from Guangzhou RiboBio Co, Ltd (Guangzhou, Guangdong, China). The Wip1-overexpression plasmid was generated by inserting Wip1 cDNA lacking its 3'-UTR into a pCMV vector. This plasmid was chemically produced by Shanghai GenePharma Co., Ltd (Shanghai, China). The siRNA-Wip1 (si-Wip1) was obtained from Qiagen GmbH (Hilden, Germany) and used to knock down the endogenous expression of Wip1. A control siRNA (si-NC) served as a negative control for si-Wip1. RNA oligonucleotides and plasmid were transfected into cells using Lipofectamine 2000 (Invitrogen; Thermo Fisher Scientific, Inc, Waltham, MA, USA) following the manufacturer's protocol.

### RNA extraction and reverse transcription-quantitative polymerase chain reaction (RT-qPCR)

TRIzol reagent (Invitrogen; Thermo Fisher Scientific, Inc.) was utilized for the isolation of total RNA from tissue specimens and cells. The concentration of total RNA was determined with a Nanodrop 2000 (Thermo Fisher Scientific, Waltham, MA). Total RNA was reversely transcribed into cDNA using the miScript Reverse Transcription kit (Qiagen GmbH, Hilden, Germany). After that, quantitative PCR (qPCR) was carried out to detect miR-129-2-3p expression with the miScript SYBR Green PCR kit (Qiagen GmbH). For the determination of Wip1 mRNA, reverse transcription was carried out using the PrimeScript RT reagent kit (Takara Bio, Dalian, China). Next, qPCR was conducted through a SYBR Premix Ex Taq™ Kit (Takara Bio) and an Applied Biosystems 7500 Real-time PCR System (Thermo Fisher Scientific, Waltham, MA). U6 served as the internal reference for miR-129-2-3p, and GAPDH was the internal control for Wip1. Relative gene expression was analyzed using the 2^-ΔΔCq^ method 19.

### Cell Counting Kit-8 (CCK-8) assay

Transfected cells were plated into 96-well plates at a density of 3×10^3^ cells per well. Five replicate wells were used for every group. After culture for 0, 24, 48, and 72 h, CCK-8 assay was carried out by inoculating 10 μL of CCK-8 reagent (Dojindo Molecular Technologies, Inc., Kumamoto, Japan) into each well. Cells were incubated at 37 °C with 5% CO_2_ for additional 2 h, and the absorbance in each well was measured using a spectrophotometric plate reader (Infinite® 200 PRO; Tecan Group, Ltd., Mannedorf, Switzerland).

### Transwell cell invasion assay

Transwell chamber (Costar™; Corning, Inc., Corning, NY, USA) precoated with Matrigel (BD Biosciences, San Jose, CA) was applied to assess the cellular invasive capacity. In detail, the transfected cells were harvested after 48 h of incubation and then suspended in FBS-free DMEM medium. In total, the upper chambers were filled with 200 μL suspension containing 5 × 10^4^ cells, while the bottom chambers were added with 500 μL of DMEM containing 20% FBS. After incubation for 24 h, the cells that invaded through the membrane were fixed with 4% paraformaldehyde and stained by 0.5% crystal violet. Finally, the non-invaded cells were cleared, and the invaded cells were photographed under an inverted microscope (Olympus IX83; Olympus Corporation, Tokyo, Japan). Five random fields of each chamber were selected for quantification.

### Tumor xenograft assay

miR-129-2-3p mimics or miR-NC-transfected cells were subcutaneously injected into the upper flank of 4-5 weeks old nude mice (Shanghai Laboratory Animal Center; Shanghai, China).The size of tumor xenograft was read every week. All of the nude mice were sacrificed four weeks after seeding. The tumor xenografts were resected and weighed. Tumor volume was analyzed using the following formula: tumor volume (mm3) = (length × width^2^)/2. All experimental procedures were approved by the Ethics Committee of Hunan Provincial People's Hospital/The First Affiliated Hospital of Hunan Normal University.

### Bioinformatics analysis and luciferase reporter assay

TargetScan 7.1 (http://www.targetscan.org/) and miRanda (http://www.microrna.org) were employed to search for the putative targets of miR-129-2-3p. The 3'-UTR regions of human Wip1 gene containing the predicted wild-type (wt) or mutant (mut) miR-129-2-3p binding sites were synthesized by Shanghai GenePharma Co., Ltd. The 3'-UTR fragments were then inserted into the pMIR-REPORT vector (Promega, Madison, WI, USA) to generate the reporter plasmids, pMIR-Wip1-3'-UTR-wt and pMIR-Wip1-3'-UTR-mut. Luciferase reporter assay was performed as follows: cells were inoculated into 24-well plates, then co-transfected with miR-129-2-3p mimics or miR-NC and pMIR-Wip1-3'-UTR-wt or pMIR-Wip1-3'-UTR-mut, using Lipofectamine 2000. Following 48 h incubation, transfected cells were collected, and the luciferase activity was determined using a dual-luciferase reporter assay system (Promega, Madison, WI, USA). The firefly luciferase activity was normalized to Renilla luciferase activity.

### Protein extraction and western blot analysis

Tissues or cells were solubilized using the active protein extraction kit (KGP1050; Nanjing KeyGen Biotech Co., Ltd., Nanjing, China) supplemented with protease inhibitors (Millipore, Billerica, MA). The concentration of total protein was detected by using an Enhanced BCA Protein Assay Kit (Beyotime Institute of Biotechnology, Shanghai, China). Equivalent of total protein was loaded for SDS-PAGE on 10% polyacrylamide gels, followed by transferring to PVDF membranes (Millipore, Billerica, MA) and blocking with 5% skimmed milk for 2 h. The membranes were then incubated overnight at 4°C with primary antibodies against Wip1 (cat. no. sc-517220; Santa Cruz Biotechnology, CA, USA) or GAPDH (cat. no. ab125247; Abcam, Cambridge, MA, USA). Subsequently, the membranes were incubated with horseradish peroxidase-conjugated immunoglobulin G goat anti-mouse (cat. no. ab6789; 1:5,000 dilution; Abcam) secondary antibodies at room temperature for 2 h. Immunoreactivity was visualized by using an enhanced chemiluminescence reagents (ECL; Pierce; Thermo Fisher Scientific, Inc.).

### Statistical analysis

All the data were expressed as mean values ± standard deviation, and subjected to Social Sciences (SPSS) version.16.0 (SPSS, Inc., Chicago, IL, USA) for data analysis. Student's t-test was used to compare the difference between two groups. Comparison among multiple groups was performed using a one-way analysis of variance (ANOVA) test followed by a Bonferroni's post hoc test. The correlation between miR-129-2-3p and clinicopathological characteristics in ICC patients was assessed using chi-square test. Spearman's correlation analysis was adopted to analyze the correlation between miR-129-2-3p and Wip1 mRNA levels in ICC tissues. The differences were defined as statistically significant if P value less than 0.05.

## Results

### miR-129-2-3p expression is decreased in ICC tissues and cell lines

**Figure 1:**
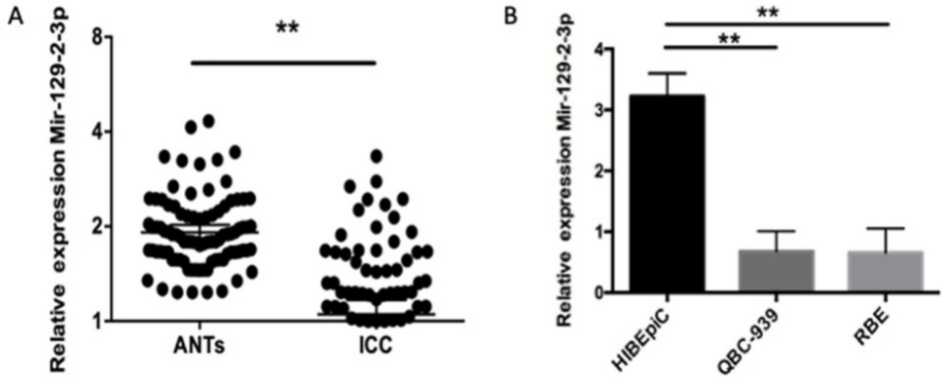
** miR-129-2-3p is downregulated in ICC tissues and cell lines.** (A) The expression level of miR-129-2-3p was assessed in 90 pairs of ICC tissues and adjacent normal tissues (ANTs) using RT-qPCR. *P<0.05 vs. ANTs. (B) RT-qPCR was carried out to measure miR-129-2-3p expression in the normal human biliary epithelial cell (BEC) and two ICC cell lines (RBE and QBC-939). **P<0.01 vs.BEC.

First of all, RT-qPCR was used for the detection of miR-129-2-3p expression in ICC tissues and adjacent normal tissues (ANTs) obtained from 90 patients with ICC. miR-129-2-3p expression was notably decreased in ICC tissues when compared with that in ANTs (Figure 1A, P<0.05). The expression level of miR-129-2-3p was also determined in four ICC cell lines, including QBC-939 and RBE. The normal human biliary epithelial cell (BEC) acted as the control. The data indicated that expression level of miR-129-2-3p was lower in the ICC cell lines than that in BEC (Figure 1B, P<0.05). A total of 90 ICC patients were subdivided into either low or high miR-129-2-3p expression groups according to the median value as a cutoff (Table 1). Lowly expressed miR-129-2-3p was frequently associated with differentiation (P=0.037) and pathology T (P=0.006). Furthermore, these data also demonstrated that miR-129-2-3p expression was dramatically associated with pathology N (P=0.042) and pathology M (P=0.01). These observations indicated that miR-129-2-3p expression may be associated with the development of ICC.

### miR-129-2-3p upregulation suppresses the proliferative and invasive abilities of ICC cells

**Figure 2:**
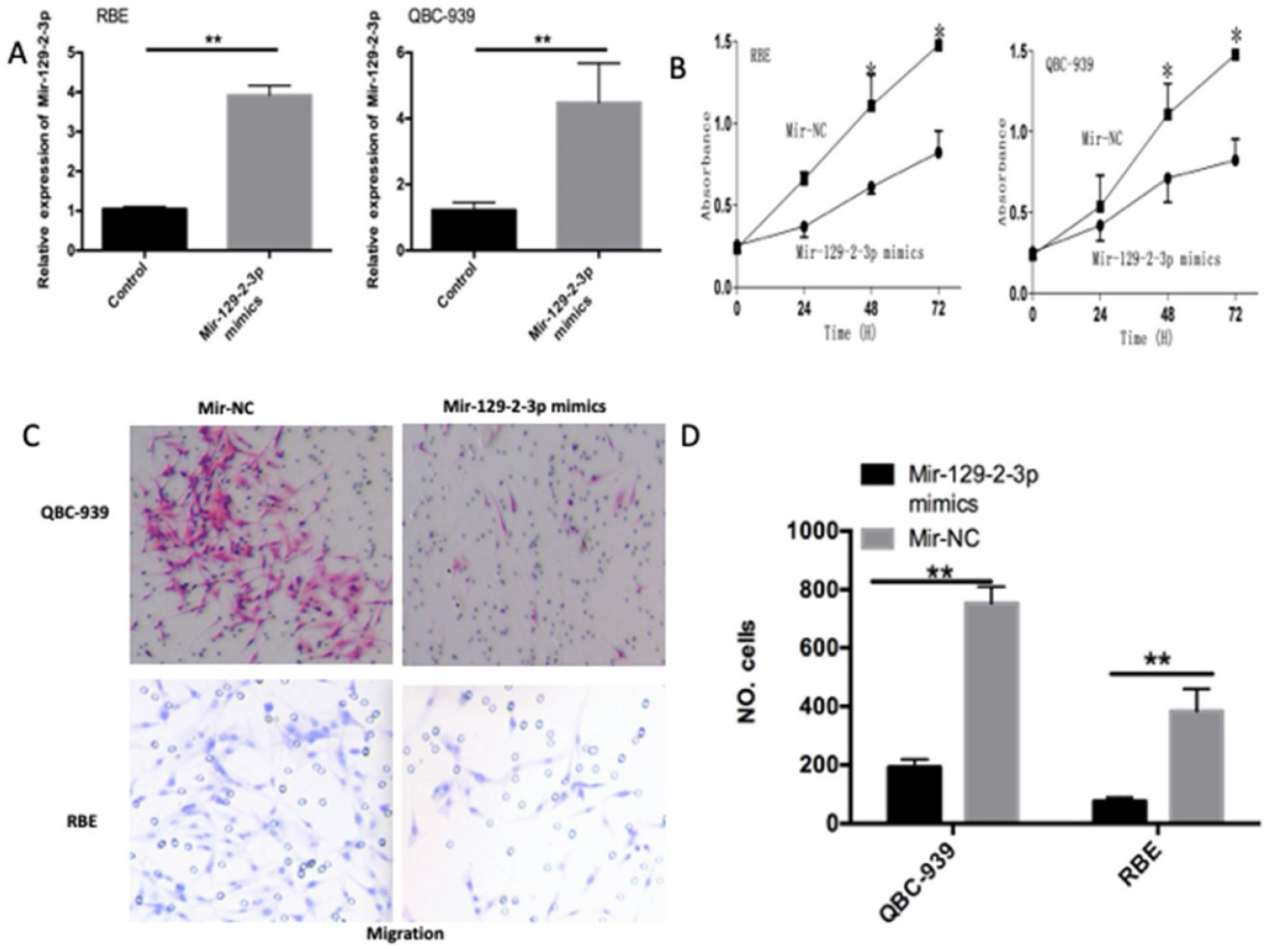
** Resumption of miR-129-2-3p expression restrains the proliferation and invasion of RBE and QBC-939 cells.** (A) The expression level of miR-129-2-3p was measured in RBE and QBC-939 cells after miR-129-2-3p mimics or miR-NC transfection. **P<0.01 vs. miR-NC. (B) CCK-8 assay was performed to determine the proliferation in miR-129-2-3p mimics or miR-NC-transfected RBE and QBC-939cells. *P<0.05 vs. miR-NC. (C, D) The effect of miR-129-2-3p upregulation on RBE and QBC-939cell invasion was explored using transwell cell invasion assay. **P<0.01 vs. miR-NC.

To investigate the functions of miR-129-2-3p in ICC progression, we transfected the miR-129-2-3p mimics into QBC-939 and RBE cells and then transfection efficiency was evaluated using RT-qPCR (Figure 2A, P<0.05). CCK-8 and transwell cell invasion assays were utilized to determine the differences between QBC-939 and RBE cells after transfection with miR-129-2-3p mimics or miR-NC. The miR-129-2-3p overexpressing-QBC-939 and RBE cells exhibited weaker proliferative (Figure 2B, P<0.05) and invasive (Figure 2C, P<0.05) abilities in comparison with that in cells transfected with miR-NC. These data implied that miR-129-2-3p exerts tumor suppressive roles in ICC by decreasing cell proliferation and invasion *in vitro*.

### Wip1 is a direct target gene of miR-129-2-3p in ICC cells

**Figure 3:**
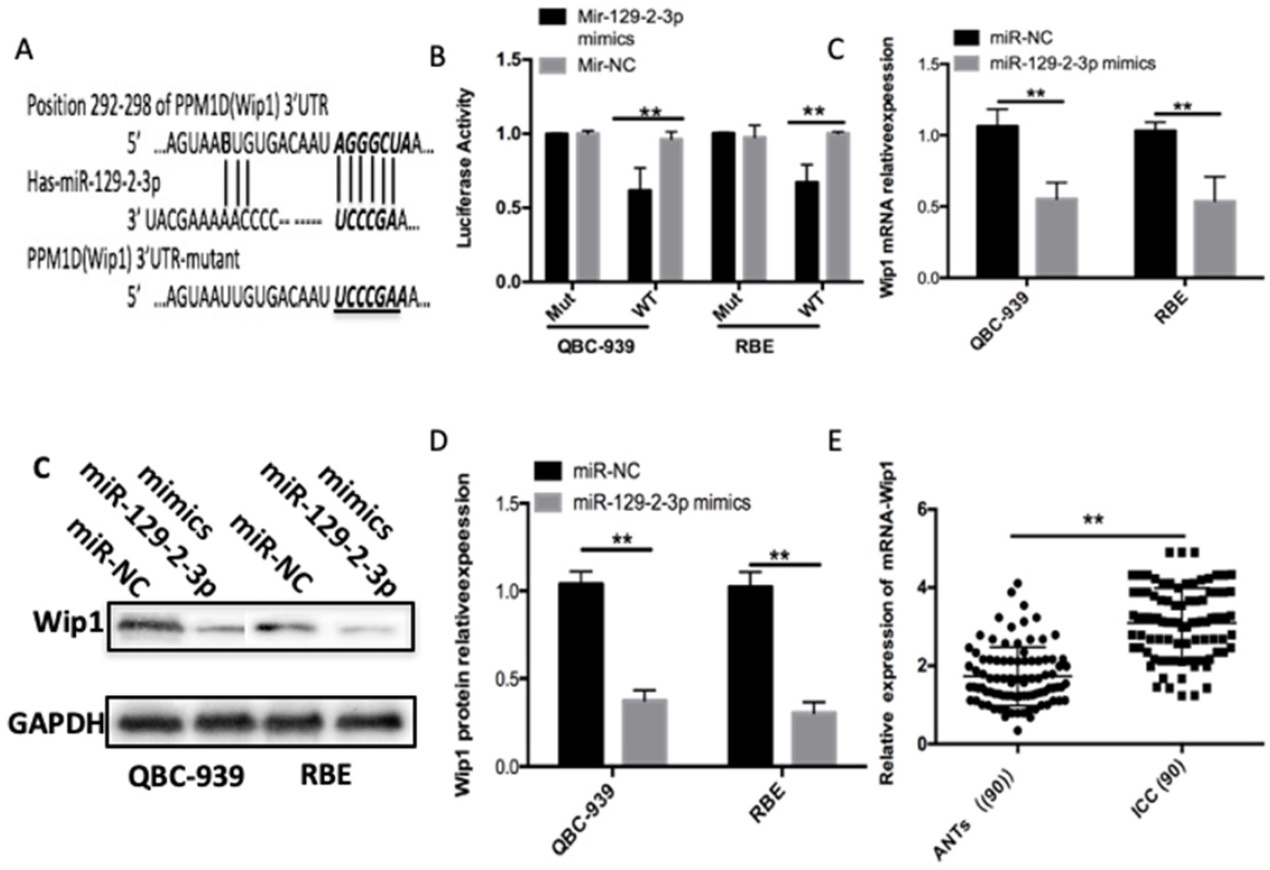
** Wip1 is a direct target gene of miR-129-2-3p in ICC cells.** (A) The 3'-UTR of the Wip1 contains a potential miR-129-2-3p binding site. The mutant 3'-UTR region of the Wip1 is also shown. (B) pMIR-Wip1-3'-UTR-wt or pMIR-Wip1-3'-UTR-mut along with miR-129-2-3p mimics or miR-NC was introduced into RBE and QBC-939cells. After 48 h culture, luciferase reporter assay was utilized for the measurement of the luciferase activity. **P<0.01 vs. miR-NC. (C, D) RBE and QBC-939cells were transfected with miR-129-2-3p mimics or miR-NC. The mRNA and protein levels of Wip1 were measured by RT-qPCR and western blot analysis, respectively. *P<0.05 vs. miR-NC. (E) The mRNA expression of Wip1 was detected in 90 pairs of ICC tissues and ANTs using RT-qPCR. **P<0.01 vs. ANTs.

To gain insight into the mechanisms through which miR-129-2-3p modulates the proliferation and invasion of ICC cells, bioinformatics analysis was performed to predict the putative targets of miR-129-2-3p and found that the 3′-UTR of Wip1 contains a highly conserved binding site for miR-129-2-3p (Figure 3A). Luciferase reporter assay was carried out to explore whether miR-129-2-3p was able to directly bind to the 3'-UTR of Wip1 in ICC cells. Resumption of miR-129-2-3p expression significantly suppressed the luciferase activity of pMIR-Wip1-3'-UTR-wt in QBC-939 and RBE cells (P<0.05); however, the inhibitory effect was not observed in cells with pMIR-Wip1-3'-UTR-mut (Figure 3B). We next increased miR-129-2-3p expression in QBC-939 and RBE cells to investigate whether the expression levels of Wip1 were altered in response. Introduction of miR-129-2-3p evidently reduced Wip1 mRNA (Figure 3C, P<0.05) and protein (Figure 3D, P<0.05) expression levels in QBC-939 and RBE cells. Furthermore, we detected Wip1 expression in ICC tissues and ANTs, revealing that expression level of Wip1 mRNA was higher in ICC tissues than that in ANTs (Figure 3E, P<0.05). Taken together, Wip1 is a direct target gene of miR-129-2-3p in ICC cells.

### Silenced Wip1 expression restricts cell proliferation and invasion in ICC

**Figure 4:**
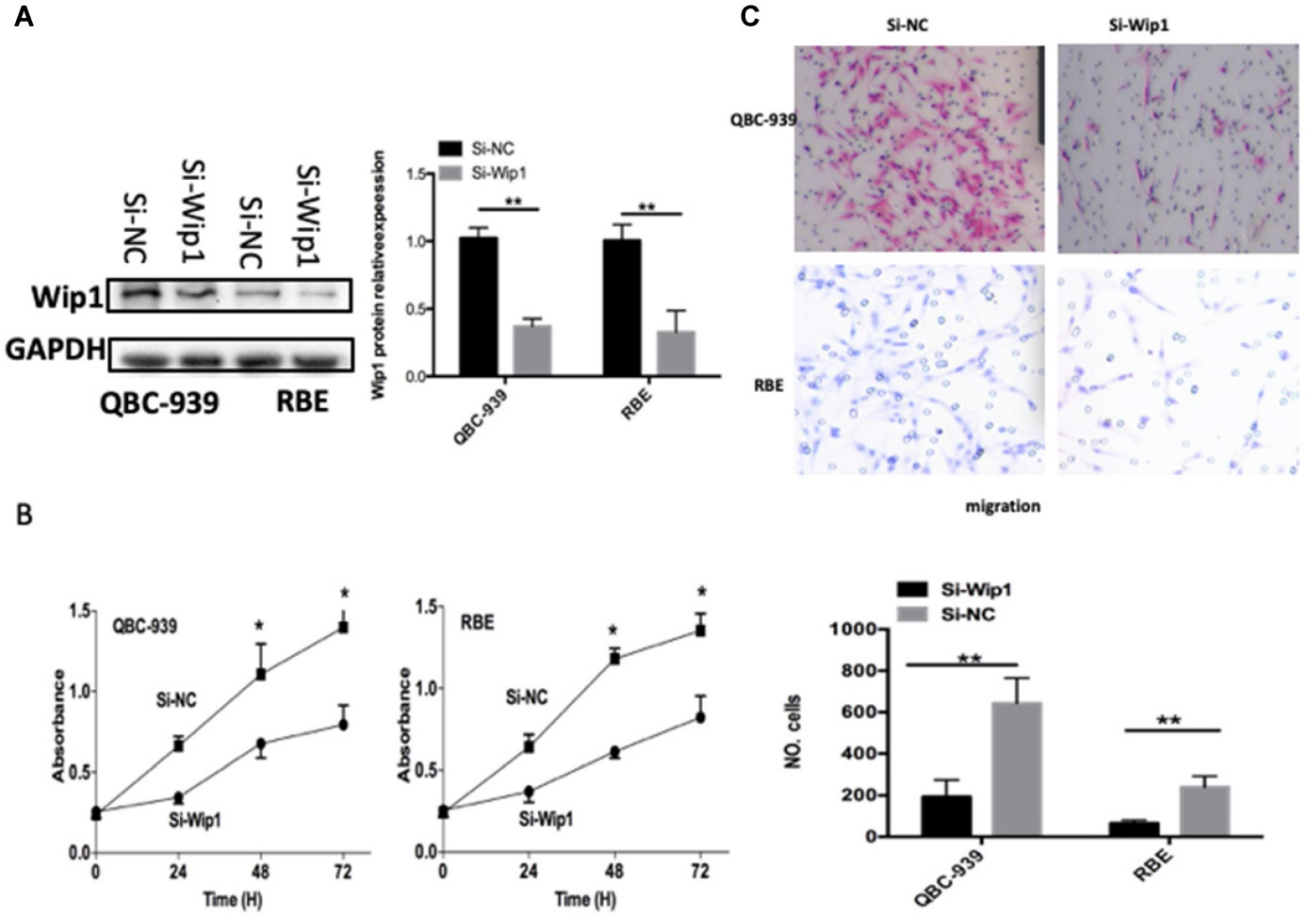
** Knockdown of Wip1 suppresses the proliferation and invasion of RBE and QBC-939cells. RBE and QBC-939cells were transfected with si-Wip1 or si-NC.** (A) 72 h after transfection, the protein level of Wip1 was determined by western blot analysis. **P<0.01 vs. si-NC. (B, C) The proliferation and invasion was assessed by CCK-8 and transwell cell invasion assays, respectively. Wip1-silencing evidently inhibited the abilities of proliferative and invasive in RBE and QBC-939cells. **P<0.01 vs. si-NC.

To explore the functions of Wip1 in OS, si-Wip1 was utilized to silence endogenous Wip1 expression in QBC-939 and RBE cells. The protein level of Wip1 was evidently silenced in QBC-939 and RBE cells after si-Wip1 transfection, as revealed by western blot analysis (Figure 4A, P<0.05). The results of CCK-8 and transwell cell invasion assays indicated that the proliferation (Figure 4B, P<0.05) and invasion (Figure 4C, P<0.05) of QBC-939 and RBE cells was suppressed after Wip1 depletion. Consequently, silencing Wip1 expression exhibited similar effects as miR-129-2-3p overexpression in ICC cells, further suggesting Wip1 as a function target of miR-129-2-3p in ICC cells.

### Wip1 restoration markedly rescues the miR-129-2-3p-mediated inhibition of cell proliferation and invasion in ICC

**Figure 5:**
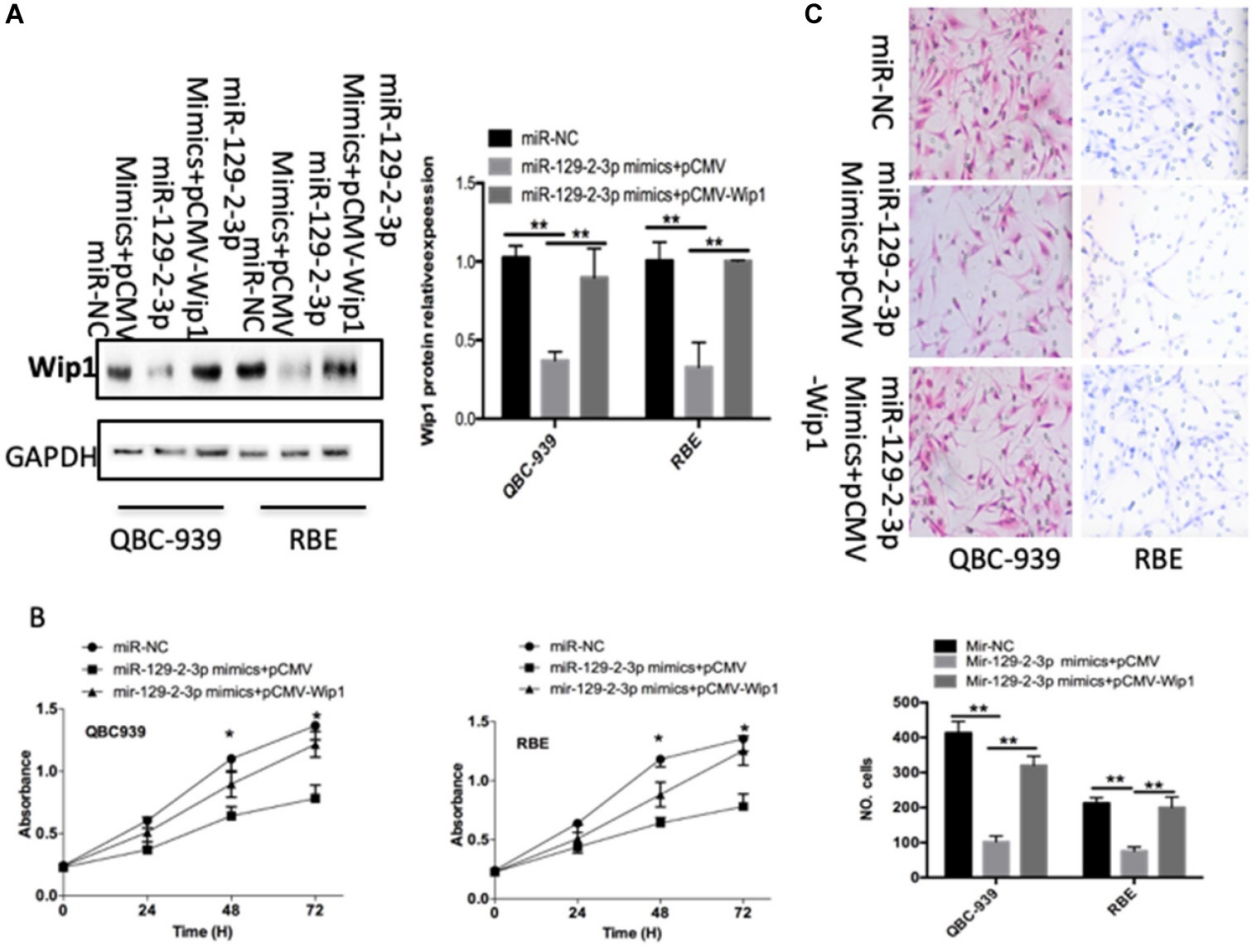
** Wip1 is required for miR-129-2-3p-directed inhibition of RBE and QBC-939cell proliferation and invasion. miR-129-2-3p-overexpressing RBE and QBC-939cells were transfected with pCMV-WIP1 or pCMV.** (A) Transfected cells were collected after 72 h of incubation and subjected to western blot analysis for Wip1 protein expression. **P<0.01 vs. miR-NC. **P<0.01 vs. miR-129-2-3p mimics+pCMV. (B, C) The proliferation and invasion of RBE and QBC-939cells treated as described was investigated by CCK-8 and transwell cell invasion assays, respectively. **P<0.01 vs. miR-NC. **P<0.01 vs. miR-129-2-3p mimics+pCMV.

To further clarify whether decreasing Wip1 expression by miR-129-2-3p upregulation was responsible for the restriction of ICC cell proliferation and invasion, we restored Wip1 expression in miR-129-2-3p overexpressing-QBC-939 and RBE cells via cotransfection with Wip1 overexpression plasmid pCMV-Wip1 (Figure 5A, P<0.05). In addition, functional experiments revealed that the inhibitory effects of miR-129-2-3p upregulation in QBC-939 and RBE cell proliferation (Figure 5B, P<0.05) and invasion (Figure 5C, P<0.05) were partially rescued by Wip1 restoration. In summary, Wip1 is a downstream mediator in the tumor suppressive roles of miR-129-2-3p in ICC cells.

### miR-129-2-3p impairs the ICC tumor growth *in vivo*

**Figure 6:**
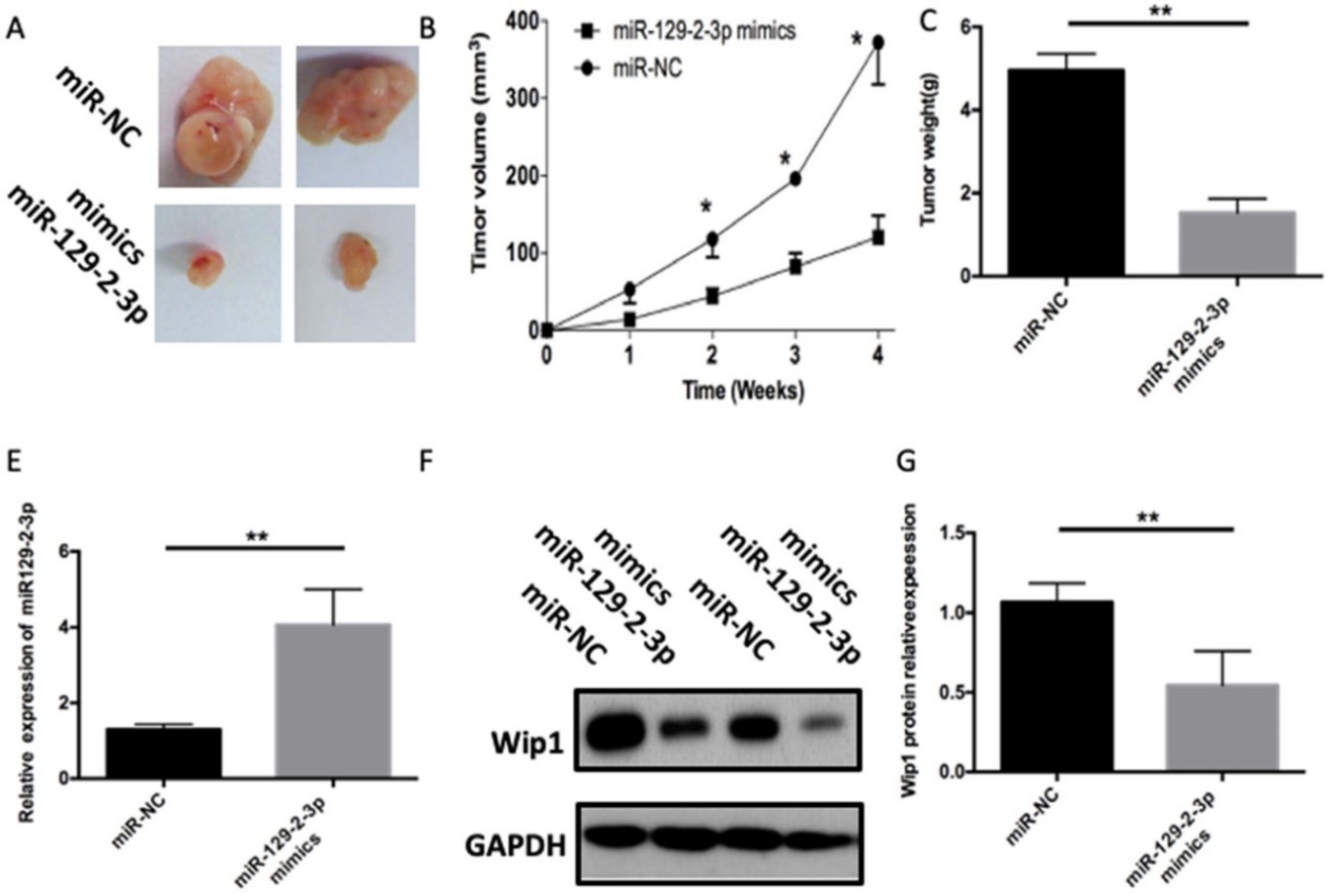
** miR-129-2-3p upregulation impairs ICC tumor growth *in vivo.*** (A) Representative images of tumor xenografts derived from QBC-939 cells transfected with miR-129-2-3p mimics or miR-NC. (B) The volume of tumor xenografts in the miR-129-2-3p mimics group was smaller than that in the miR-NC group. *P<0.05 compared with miR-NC. (C) The tumor xenografts in the miR-129-2-3p mimics and miR-NC group were excited and weighed after four weeks of implantation. **P<0.01 vs. miR-NC. (D) RT-qPCR was performed to analyze miR-129-2-3p expression in the tumor xenografts. **P<0.01 vs. miR-NC. (E) The expression level of Wip1 protein in tumor xenografts was analyzed by western blot analysis. **P<0.01vs. miR-NC.

Tumor xenograft assay was performed to assess the influence of miR-129-2-3p on cell tumorigenicity *in vivo*. ICC cells transfected with miR-129-2-3p mimics were inoculated into nude mice, and miR-NC-transfected cells were used as a control. The volume and weight of tumor xenograft derived from miR-129-2-3p mimics-transfected ICC cells was notably smaller (Figures 6A and B, P<0.05) and lighter (Figure 6C, P<0.05) than that in the miR-NC groups. Next, RT-qPCR was utilized for the detection of miR-129-2-3p expression in the tumor xenograft. Higher miR-129-2-3p expression was observed in the tumor xenograft of miR-129-2-3p mimics group compared with that in the miR-NC group (Figure 6D, P<0.05). Furthermore, the protein level of Wip1 in the tumor xenograft was determined via western blot analysis. Results revealed that the expression of Wip1 protein was evidently downregulated in the miR-129-2-3p mimics-treated nude mice group (Figure 6E, P<0.05). These results implied that miR-129-2-3p impaired the tumor growth of ICC cells *in vivo*.

## Discussion

Intrahepatic cholangiocarcinoma (ICC) is the second most common primary hepatic malignancy with poor prognosis 17, 20. Despite improvements in its diagnosis and therapy, the prognosis for ICC patients remains poor. An improved understanding of ICC tumorigenicity and consequential identification of novel therapeutic targets would improve the prognosis of ICC patients 17. MicroRNAs (miRNAs) are a class of highly conserved, small endogenous non-coding RNAs that regulate gene expression at the posttranscriptional level and participate in important cellular processes, including development, apoptosis, proliferation, differentiation, metabolism 9,21. Several studies have demonstrated downregulated expression of miRNAs in ICC tissues and cell lines, in which these miRNAs exert important roles in ICC apoptosis, cell proliferation, invasion and migration 9,21. In this paper, we elucidate the potential role of miR-129-2-3p in the pathogenesis of ICC and investigate the possibilities of using miR-129-2-3p as diagnostic and prognostic marker, as well as therapeutic target in ICC.

The miR-129 family includes two precursors miR-129-1 and miR-129-2 which are processed to three mature miRNAs, miR-129-5p, miR-129-1-3p and miR-129-2 21. Few studies have investigated the roles of the miR-129 family members in cell proliferation and metastasis in ICC. Dual functions for miR-129, as a tumor suppressor and oncogene, have been confirmed in various types of carcinomas. miR-129-5p has been reported to be downregulated in neuroendocrine tumors, prostate cancer, lung cancer and gastric cancer and functions as a tumor-suppressor role in these carcinomas 22-24. Epigenetic regulation of miR-129-2 was demonstrated in glioma and lung cancer 25,26. In contrast, however, miR-129 is also upregulated in several solid tumors and non-cancerous tissues from cancer patients with lymph node metastases 27-29.

Our previous findings indicate that Wip1 is involved in the tumorigenicity and invasion of human ICC at least in part through the MMP-2 signaling pathway 17. Our further data show that both iNOS and Wip1 promote ICC cell migration and invasion by up-regulating MMP expression 18. To evaluate the underlying mechanism, we find miR-129-2-3p directly targets wip1 to suppress the proliferation and invasion of intrahepatic cholangiocarcinoma.

Wip1, located on chromosome 17q22-q23, acts as an oncogene and inhibits p53 activity when expressed at high levels along with oncogenes 30. These features are mainly connected with Wip1 ability to regulate DNA damage response (DDR) signaling and MAPK kinases pathway p53 network, including p38, p53, ATM, Chk2, and γ-H2AX 10,31,32. Accumulating studies identified that high Wip1 expression disrupted the homeostasis maintained by the p38 MAPK-p53-Wip1 pathway, which caused downstream Wnt-p53 inactivation through p38 MAPK dephosphorylation, and promoted the development of malignant in humans by reducing p16 protein levels 33,34. Our previous data have indicated that Wip1 is oncogenic and is involved in invasive growth in renal cancer cells and ICC cells 12,17. Our data also show Wip1 involved in the tumorigenicity and invasion of human ICC in part through the MMP-2 signaling pathway 17. In this study, we found that miR-129-2-3p was the upstream regulator of Wip1.

In summary, our results revealed that miR-129-2-3p expression was decreased in ICC tissues and cells. A low miR-129-2-3p expression was significantly correlated with malignant clinical features of patients with ICC. Additionally, we have shown, for the first time, that miR-129-2-3p exerted tumor suppressive roles in ICC progression through its target gene Wip1. Hence, this study provided functional evidence that fully supports the hypothesis that miR-129-2-3p might be a promising target for the management of patients with ICC.

## Figures and Tables

**Table 1:** Clinicopathological correlation with miR-129-2-3p expression

Variable	miR-129-2-3p		P
	Low n = 74	high, n = 16	
Age (Y) (<50/≥50)	29/45	5/11	0.553
M/F	42/32	10/6	0.673
Complicated bile duct stone+/-	38/36	3/13	0.65
Tumor size (cm) (<5/≥5)	35/39	13/3	0.297
Well/moderate/poorly differentiation	12/23/39	10/4/2	0.037
Pathology T (T1+2)/(T3+4)	19/55	10/6	0.006
Pathology N 0/1	21/53	14/2	0.042
Pathology M 0/1	19/55	13/3	0.01
